# Descriptive analysis of TikTok content on vaccination in Arabic

**DOI:** 10.3934/publichealth.2025010

**Published:** 2025-01-17

**Authors:** Malik Sallam, Kholoud Al-Mahzoum, Lujain Alkandari, Aisha Shabakouh, Asmaa Shabakouh, Abiar Ali, Fajer Alenezi, Muna Barakat

**Affiliations:** 1 Department of Pathology, Microbiology and Forensic Medicine, School of Medicine, The University of Jordan, Amman 11942, Jordan; 2 Department of Clinical Laboratories and Forensic Medicine, Jordan University Hospital, Amman 11942, Jordan; 3 School of Medicine, The University of Jordan, Amman 11942, Jordan; 4 Department of Clinical Pharmacy and Therapeutics, Faculty of Pharmacy, Applied Science Private University, Amman 11931, Jordan

**Keywords:** health communication, misinformation, social media, vaccination, content quality

## Abstract

The extensive impact of social media on communication of public health information is a growing concern. This is particularly worrying in the context of vaccination. Thus, we investigated the quality of TikTok videos regarding vaccination in Arabic, with examination of the association of video source and content type with the information quality and video engagement metrics. The final sample comprised a total of 129 TikTok videos in Arabic posted between January 2021 and July 2024. Videos were categorized based on the source [healthcare professional (HCPs), lay individuals, media], and content type (COVID-19 vaccination, childhood vaccination, general vaccination, others). We utilized a miniaturized version of the DISCERN instrument (mini-DISCERN) scale to evaluate information quality by two independent raters and assessed video engagement metrics (Likes, Comments, Shares, and Saves). The results indicated a statistically significant discrepancy in information quality, with videos from HCPs and media outlets scoring higher on the mini-DISCERN scale compared to those from lay individuals [mean: (4.818 ± 0.726) vs. (4.053 ± 1.441) vs. (2.003 ± 1.640), *P* < 0.001]. The highest information quality was found for videos on childhood vaccination, whereas content on COVID-19 vaccination was rated significantly lower on mini-DISCERN [mean: (4.510 ± 1.269) vs. (2.542 ± 1.827), *P* < 0.001]. Videos with higher engagement metrics, particularly those from lay individuals, were negatively correlated with information quality. Linear regression analysis confirmed the significant influence of the creator background (β = −0.618, *P* < 0.001) and video topic (β = 0.179, *P* = 0.009) on information quality. This study highlights the critical role of content creator background and topic on the quality of vaccination-related information on TikTok in Arabic. We emphasize the need for stringent verification of TikTok content, especially from lay individuals, as videos with higher engagement metrics often contained lower-quality information regarding vaccination. We recommend enhanced support for content from HCPs and targeted digital literacy programs to combat vaccine misinformation on TikTok effectively.

## 1. Introduction

The growth and widespread use of social media platforms have significantly changed the dynamics of health-related communication [1]–[3]. The social media platforms have dramatically transformed the way health information is shared, discussed, and received by the public [4]. This shift affects every aspect of health communication from public health messaging and awareness campaigns to individual health decisions and discussions [5]. Additionally, social media platforms have been shown to affect the public perceptions and behaviors regarding health attitudes and behaviors, including vaccination [6].

Research has highlighted the increasing impact of social media on public health communication, particularly during the COVID-19 pandemic [7]–[11]. For example, De Rosis *et al*. demonstrated how social media platforms like Twitter can capture real-time public reactions and emotions [12]. De Rosis *et al*. also highlighted the utility of social media in aligning public sentiment with health measures and its applicability in shaping health messaging [13]. Similarly, Lopreite *et al*. identified the role of social media as an early warning system, successfully predicting COVID-19 hotspots across Europe [14]. Collectively, these researchers emphasized the importance of integrating digital surveillance into public health systems [14]–[16]. Furthermore, Parisi *et al*. focused on vaccine skepticism in TikTok, showing that discussions on this social media platform were largely centered on safety concerns [17]. Parisi *et al*. concluded that the presence of healthcare professionals (HCPs) on TikTok contributed to less polarization, with subsequent positive value for vaccine communication [17]. Together, the aforementioned studies highlighted the key role of social media in disseminating health information and monitoring public sentiment during health crises.

TikTok is a social media platform known for its short, engaging video format [18]. In less than 10 years since its development in 2016 by ByteDance (https://www.bytedance.com/en/, accessed on 24 August 2024), TikTok rapidly emerged as a popular source for disseminating health-related information on various topics [19]–[23]. Evidence suggests that lay individuals increasingly turn to TikTok for health information [20],[24],[25]. As suggested by Song *et al*., this is related to TikTok platform design features which effectively satisfy the users basic psychological needs for autonomy, relatedness, and competence; therefore, TikTok is increasingly perceived as an appealing source for engaging and understanding health-related content [26],[27].

The wide reach nature of TikTok content (live feeds and videos) enables swift information spread [28]. The recommendation system of TikTok combines both collaborative and content-based filtering to personalize content, which enables even users with few followers to reach wide audiences [29]–[31]. However, concerns about algorithmic transparency on TikTok raise issues of user control and potential biases in content promotion [32].

TikTok content can be particularly influential during health crises, which has been shown during the coronavirus disease 2019 (COVID-19) pandemic, and the recent monkeypox (Mpox) global outbreak [33]–[35]. However, alongside its potential benefits, TikTok content also poses significant challenges due to its potential to spread misinformation [36],[37]. Misinformation refers to inaccurate or misleading information that contradicts well-established scientific evidence and expert consensus, often lacking support from credible scientific research results [38],[39]. The spread of misinformation on social media platforms including TikTok can quickly gain momentum, leading to widespread public health issues and undermining efforts to manage health crises effectively [35],[40]. This was shown in a study on COVID-19 vaccination by Berdida *et al*., which demonstrated that TikTok comments by the people of the Philippines often reflected a reluctance to get vaccinated, driven by personal reasons, doubts about vaccine effectiveness, and fears of severe side effects [41].

In the context of COVID-19 vaccine misinformation, a comprehensive study conducted by Morgan Lundy revealed several key insights [42]. In the seminal study, Lundy concluded that the unique features of TikTok, such as reusable audio and interactive capabilities, offer new paths for the dissemination of misinformation [42]. Importantly, Lundy found that TikTok users commonly employ coded language, intentional misspellings, and alternative hashtags to bypass anti-misinformation measures [42]. Common vaccine misinformation themes included exaggerated side effects, doubts about vaccine production and approval, and underestimations of COVID-19 risks [42]. Moreover, van Kampen *et al*. analyzed the top 100 TikTok videos tagged with the hashtag #covidvaccine [43]. They identified the important role of HCPs in spreading accurate vaccine information, with all HCP-created videos supporting vaccine use, contributing to 81% of the videos promoting vaccination [43].

In the Arab countries of the Middle East, the propagation of misinformation and conspiracy theories has been notably prevalent during public health crises such as the COVID-19 pandemic and the recent Mpox outbreak [44]–[47]. For example, a large study that was conducted primarily in Jordan and Kuwait revealed an extensive prevalence of COVID-19 vaccine hesitancy [48]. Specifically, 28% of participants in the study believed that COVID-19 vaccines were designed to implant microchips for surveillance purposes, and 23% associated COVID-19 vaccines with infertility [48]. Furthermore, during the 2022 Mpox outbreak, a study among the general public in Jordan showed that a substantial portion of participants expressed distrust in official narratives about the virus origin, with 66% being skeptical of the provided explanations [49]. Additionally, 64% endorsed the notion that viruses could be bioweapons created by global superpowers for control purposes [49]. Collectively, several researchers highlighted the challenges faced in combating health-related misinformation in the Arab countries of the Middle East region [44],[45],[49],[50]. The impact of this misinformation extended beyond vaccine hesitancy to involve increased reporting of long COVID symptoms and a heightened perception of post-vaccination side effects in several Arab countries [51]–[53].

The growing influence of social media platforms such as TikTok on public health communication highlights the critical need for detailed analysis of their content, especially concerning vaccine misinformation within Arabic-speaking populations [5],[35]. Arab communities, dispersed across geographic regions with differing levels of access to healthcare, income, and education, may respond uniquely to misinformation, distinctively from other cultural groups as shown by Noman *et al*. [54].

As identified by Radcliffe *et al*. [55], the Middle East and North Africa (MENA) region is a leader in the social media market globally, with TikTok emerging as a major source of information. However, the TikTok platform presents significant challenges due to its algorithm that favors user engagement [56]. TikTok algorithms were reported to prioritize content that maximizes user engagement, often by promoting videos that trigger strong emotional responses [57],[58]. This economically driven design, aiming to increase user retention and platform profitability, can unintentionally amplify content that reinforces existing biases or spreads misinformation, facilitating the formation of “echo chambers” [59]–[61]. A study underlined the dangers of such misinformation dynamics on TikTok, specifically highlighting the spread of measles-rubella vaccine misinformation in Jordan [62]. In the aforementioned study, TikTok content of lower quality garnered significantly higher user engagement than content with more accurate information [62]. Therefore, the rapid dissemination of misinformation on TikTok could pose a serious public health threat [35],[63].

Given the critical lack of research on vaccine-related misinformation on TikTok within Arab countries, there is a need for systematic investigations into this area. Such studies are essential for designing targeted interventions to enhance health literacy, counteract misinformation, and strengthen public health efforts in these culturally and linguistically varied communities. Consequently, we aimed to delineate the characteristics of vaccine-related content in Arabic-language TikTok videos and assess the videos' content quality using the validated DISCERN instrument. This tool was employed by researchers who evaluated the quality of TikTok videos health content [62],[64]–[67]. This approach can provide crucial insights into the complexities of digital health communication in Arabic and suggest strategies to address the negative impact of misinformation on attitude to vaccination.

## 2. Materials and methods

### 2.1. Study design

The study was based on a descriptive, cross-sectional design to examine Arabic-language content on TikTok regarding vaccination. We implemented a pre-defined search strategy using a list of ten carefully chosen keywords to enable a comprehensive view of the TikTok content on vaccination in Arabic language. The selection of ten keywords was an *ad hoc* decision aimed at balancing the need for a comprehensive search with feasibility to ensure the search process remained focused without being overwhelming.

**Figure 1.**
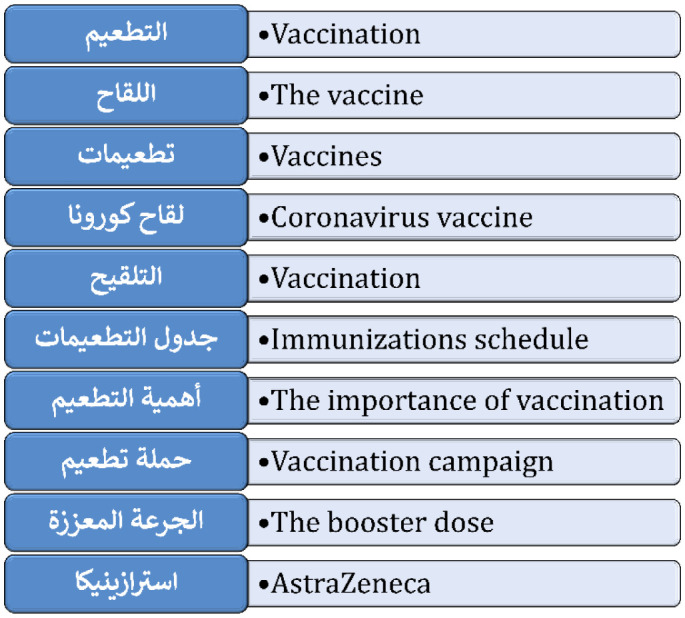
The list of original search keywords in Arabic and its corresponding translated versions in English. As noted in the first and fifth keywords, the term “vaccination” can be expressed with two synonyms in Arabic.

The list of keywords was collaboratively developed by the first and senior authors, both of whom have expertise in assessing vaccination attitudes through previous studies and publications on vaccines. For the selected keywords, we aimed to provide a concise yet comprehensive scope to capture content on general vaccine discussions, public health vaccination campaigns, vaccine hesitancy, COVID-19 vaccines, and immunization practices. The term “AstraZeneca” was specifically included due to the significant controversy in early 2024 regarding rare COVID-19 vaccination side effects, leading to widespread dissemination of both accurate and misleading information, particularly in Arabic-speaking countries [68],[69]. This approach enabled the systematic retrieval of a broad spectrum of relevant TikTok videos in Arabic, and the keywords used in the search strategy and its translation into English are detailed in (Figure 1).

### 2.2. The search strategy for retrieving relevant TikTok videos on vaccination in Arabic and videos' engagement metrics

The search strategy involved sequential manual input of the list of 10 predefined keywords into the TikTok search bar, with the search settings adjusted to filter and display only video results. To account for the variability in TikTok's search algorithms and potential geographic bias, the searches were conducted in two distinct locations: Jordan and Kuwait. The top 5 videos for each keyword were collected independently by four authors (Lujain Alkandari, Aisha Shabakouh, Asmaa Shabakouh, and Abiar Ali). Data collection occurred over a concise timeframe, from 12 July 2024 to 15 July 2024. The narrow timeframe was selected to minimize bias that would arise from fluctuating engagement dynamics on TikTok. By focusing on a narrow, defined period, we aimed to capture a relatively stable and representative snapshot of user interactions to reduce variability that could affect the analysis of engagement metrics of the analyzed content.

Then, the retrieved videos were compiled and screened for relevance with exclusion of any content not in Arabic, not related to vaccination, or duplicates and this screening process was done by the first and senior authors. Eligibility criteria were agreed upon before the search by the first and senior authors defined as Arabic-language videos that addressed aspects of vaccination, such as efficacy, safety, side effects, misinformation, or public health vaccination campaigns regardless of the time of posting or duration of the video.

For each included TikTok video, we extracted multiple features and engagement metrics to assess the content perceived impact and reach, including the date of video posting, and the number of “Likes”, “Comments”, “Saves”, and “Shares”. Information about the video's creators (number of followers and background if applicable) were also recorded. Content creators were categorized into three groups for further analysis: (1) Traditional media and journalists (including TV/news websites) and was referred to as “Media”; (2) Self-identified HCPs, including physicians, pharmacists, nurses, and doctors of pharmacy; and (3) Lay individuals, which included creators with unidentified backgrounds or those not fitting the first two categories, based on the methodological approach used previously by Abdaljaleel *et al*. [62].

### 2.3. Ethical approval of this study

This study was approved by the Institutional Review Board (IRB) at the School of Pharmacy – Applied Science Private University (Approval number: 2024–PHA–28) granted on 11 July 2024.

### 2.4. Evaluation of TikTok content quality

To evaluate the quality of health information in the TikTok videos included in this study, we employed a miniaturized version of the DISCERN instrument (mini-DISCERN), originally developed by Charnock *et al*. [70]. This validated tool was designed to objectively assess the quality and reliability of written health information and has been employed to assess the quality of health information of TikTok videos in contexts including those related to vaccination [22],[62],[64]–[67],[71]–[74]. For our objectives, the instrument was adapted to include three items that evaluate critical aspects of informational quality: Credibility and unbiased nature of content, clarity, and scientific accuracy.

Each item on the mini-DISCERN was rated on a scale from 1 (poor quality) to 5 (excellent quality), and the exact items were: (1) Is the video balanced and unbiased? (2) Does the video scientifically describe the benefits/risks of vaccination? and (3) Does the video contain clear misinformation regarding vaccination? Here, we operationalized the term “misinformation” by identifying content that included demonstrably false or unsupported claims about vaccines, such as those related to safety, efficacy, or conspiracy theories [38],[39].

The evaluation process was conducted independently by two raters with expertise in vaccination (the first and senior author): A Consultant in Clinical Virology and an Associate Professor in Clinical Pharmacy and Therapeutics, respectively. To ensure the reliability of the assessments, we used the Cohen kappa (κ) statistic to measure the agreement between the two raters and to confirm consistency in the evaluations. The overall mini-DISCERN score for each video was then calculated by averaging the sum of the ratings for the three items divided by three. We further categorized the overall average mini-DISCERN scores into two groups: Lower quality (1.00–3.00) and higher quality (3.01–5.00), in order to simplify the analysis and interpretation of results.

### 2.5. Statistical analysis

All statistical analyses were conducted using IBM SPSS Statistics for Windows, Version 26.0 (Armonk, NY, USA: IBM Corp). The association between categorical independent variables (such as the source of TikTok content) and scale-dependent variables (such as video engagement metrics and the mini-DISCERN score) was analyzed using non-parametric tests. This statistical choice was based on the non-normal distribution of the scale variables as confirmed by the Kolmogorov-Smirnov test (*P* < 0.001). The Kruskal-Wallis test (K-W) was employed for comparisons across more than two groups, while the Mann-Whitney *U* test (M-W) was used for two-group analyses.

To assess the reliability of the content quality evaluations between the two raters, the Cohen's kappa (κ) statistic was utilized. The κ values were classified to interpret the level of agreement as follows: Less than 0.20 indicated poor agreement, 0.21–0.40 fair, 0.41–0.60 good, 0.61–0.80 very good, and 0.81–1.00 excellent agreement [75]. To account for variations in the number of followers per content creator, the engagement metrics (Likes, Comments, Shares, and Saves) were adjusted by calculating a weight for each video as the inverse of the follower count (weight = 1/number of followers). These weights were then applied to each engagement metric to generate weighted values (e.g., weighted Likes = Likes × weight) based on the approach used previously by Abdaljaleel *et al*. [62]. Kendall's Tau (τb) bivariate correlations were used to examine the relationships between the mini-DISCERN scores and weighted engagement metrics. Linear regression analysis was conducted to identify factors associated with varying levels of mini-DISCERN scores, categorized into lower (1.00–3.00) and higher (3.01–5.00). A significance level of *P* < 0.050 was set for all statistical tests to determine relevance.

## 3. Results

### 3.1. Characteristics of the TikTok videos included as the final sample

**Figure 2.**
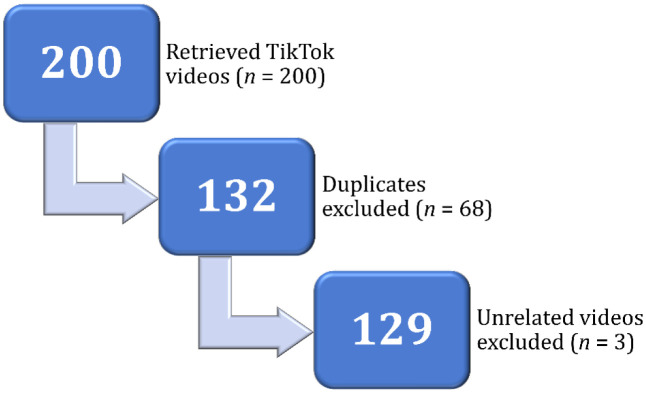
The process of selecting TikTok videos' dataset.

Of 200 videos, three videos were unrelated to the topic of vaccination in humans (one on veterinary vaccines and two were on future epidemics/outbreaks), and 68 videos were duplicates. Consequently, the final study sample of TikTok videos comprised 129 unique videos (Figure 2).

**Figure 3.**
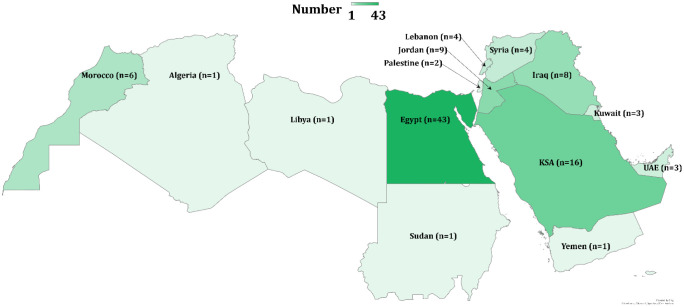
The distribution of TikTok videos included in the study based on the dialect. UAE: United Arab Emirates; KSA: Kingdom of Saudi Arabia. The map was generated in Microsoft Excel, powered by Bing, © GeoNames, Microsoft, Navinfo, TomTom, Wikipedia. We are neutral with regard to jurisdictional claims in this map.

The videos were posted between 2 January 2021 and 10 July 2024. Data on the possible country source based on the dialect used in the videos was determined for 102 videos (the remaining videos contained text or were in Standard Arabic language). For those with country sources, the majority of videos were from Egypt (n = 43, 42.2%), followed by Kingdom of Saudi Arabia (KSA, n = 16, 15.7%, Figure 3).

The most common source of videos based on creators' categories was HCPs (n = 56, 43.4%), followed by videos uploaded by Lay individuals (n = 51, 39.5%), and Media (n = 22, 17.1%). For the topics discussed in the included videos, the most common topic was COVID-19 vaccination (n = 59, 45.7%) followed by childhood vaccination (n = 35, 27.1%). The overall features of the included videos are illustrated in (Table 1).

### 3.2. Characteristics of the TikTok videos stratified per engagement metrics

**Figure 4.**
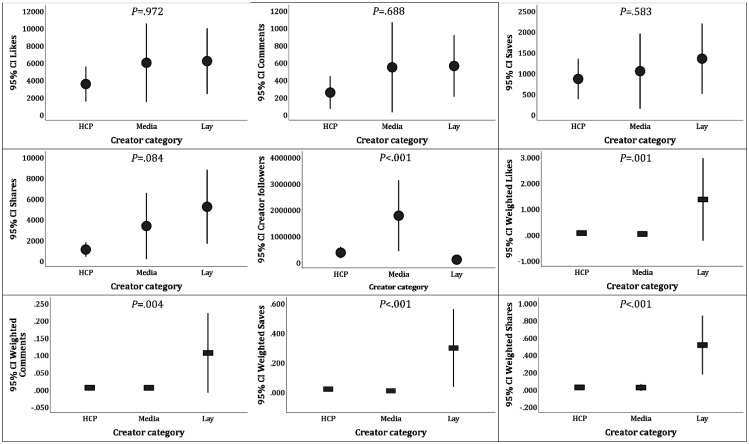
Engagement metrics and weighted engagement categories based on the number of followers of the included TikTok videos stratified per source category. HCP: Self-identified health care professional; Media: Traditional media and journalists (including TV/news websites); Lay: lay individuals; CI: confidence interval of the mean; and *P* values were calculated using the Kruskal Wallis test.

The included videos showed a statistically significant difference per source category only for the number of followers, with the highest mean number of followers observed for the Media category (mean: 1,767,848.00 ± 3,039,026.36) followed by HCPs (mean: 362,449.88 ± 795,070.495) and last by Lay individuals (mean: 94,803.20 ± 163,018.52, *P* < 0.001, KW, Figure 3). For the absolute number of engagement metrics, no statistically significant differences were observed per source category; however, a consistently higher number of weighted engagement metrics was observed among videos posted by Lay individuals with weighted mean Likes of 1.358 ± 5.648 (compared to 0.060 ± 0.168 for HCPs and 0.024 ± 0.061 for Media, *P* = 0.001), weighted mean Comments of 0.106 ± 0.409 (compared to 0.005 ± 0.014 for HCPs and 0.005 ± 0.016 for Media, *P* = 0.004), weighted mean Saves of 0.296 ± 0.932 (compared to 0.019 ± 0.064 for HCPs and 0.007 ± 0.023 for Media, *P* < 0.001), and weighted mean Saves of 0.512 ± 1.209 (compared to 0.022 ± 0.070 for HCPs and 0.021 ± 0.081 for Media, *P* < 0.001, Figure 4).

**Table 1.** General features of TikTok videos included in the study (*N* = 129).

Variable	Category	N (%)
Year	2021	20 (15.5)
	2022	16 (12.4)
	2023	49 (38.0)
	2024	44 (34.1)
Country source based on video dialect	The source could not be determined	29 (22.1)
	Algeria	1 (0.8)
	Egypt	43 (32.8)
	UAE^a^	3 (2.3)
	Iraq	8 (6.1)
	Jordan	9 (6.9)
	Kuwait	3 (2.3)
	Lebanon	4 (3.1)
	Libya	1 (0.8)
	Morocco	6 (4.6)
	Palestine	2 (1.5)
	KSA^b^	16 (12.2)
	Sudan	1 (0.8)
	Syria	4 (3.1)
	Yemen	1 (0.8)
Creator category	HCP^c^	56 (43.4)
	Media^d^	22 (17.1)
	Lay individuals	51 (39.5)
Video topic	Childhood vaccination	35 (27.1)
	COVID-19^e^ vaccination	59 (45.7)
	Influenza vaccination	6 (4.7)
	General vaccination	9 (7.0)
	HPV^f^ vaccination	2 (1.6)
	Maternal vaccination	4 (3.1)
	Measles vaccination	5 (3.9)
	Meningococcal vaccination	1 (0.8)
	Polio vaccination	5 (3.9)
	Rabies vaccination	2 (1.6)
	Rotavirus vaccination	1 (0.8)

Note: ^a^UAE: United Arab Emirates; ^b^KSA: Kingdom of Saudi Arabia; ^c^HCP: Self-identified health care professional; ^d^Media: Traditional media and journalists (including TV/news websites); ^e^COVID-19: Coronavirus disease 2019; ^f^HPV: Human papillomavirus.

### 3.3. Quality scores for the included videos

In evaluating inter-rater reliability for the overall mini-DISCERN score across three items, we observed very good to excellent agreement between the two raters. The Cohen κ values were 0.747 [standard error (SE) = 0.047, T = 12.168, *P* < 0.001], 0.900 (SE = 0.035, T = 13.096, *P* < 0.001), and 0.923 (SE = 0.032, T = 12.361, *P* < 0.001) for the three respective mini-DISCERN items. This indicated a high level of consistency in ratings across the items, with the agreement improving in the latter two.

By taking the average sum of the scores of the two raters, an overall mini-DISCERN score was assigned to each video. Stratified per the categorical study variables, a lower mini-DISCERN score was observed for the following: Videos posted by Lay individuals as opposed to those posted by HCPs and Media [mean: (2.003 ± 1.641) vs. (4.818 ± 0.726) vs. (4.053 ± 1.441), *P* < 0.001] and the videos on COVID-19 vaccination compared to videos on childhood vaccination, general vaccination, and other vaccines [mean: (2.542 ± 1.827) vs. (4.510 ± 1.269) vs. (3.444 ± 1.895) vs. (4.705 ± 0.911), *P* < 0.001, Table 2].

**Table 2.** The overall mean mini DISCERN scores for the included TikTok videos based on various categorical study variables.

Variable and category	Overall average mini DISCERN score (Mean ± SD)	*P-*value
Year		0.153
2021	4.042 ± 1.633	
2022	4.188 ± 1.581	
2023	3.367 ± 1.900	
2024	3.371 ± 1.861	
Possible country source of video		0.107
Egypt	4.287 ± 1.464	
Iraq	3.021 ± 2.075	
Jordan	3.815 ± 1.790	
Lebanon	2.708 ± 2.029	
Morocco	3.139 ± 2.045	
KSA^a^	4.542 ± 1.230	
Syria	3.125 ± 2.166	
Creator category		<0.001
HCP^b^	4.818 ± 0.726	
Media^c^	4.053 ± 1.441	
Lay individuals	2.003 ± 1.641	
Video topic		<0.001
COVID-19^d^ vaccination	2.542 ± 1.827	
Childhood vaccination	4.510 ± 1.269	
General vaccination	3.444 ± 1.895	
Other types of vaccines^e^	4.705 ± 0.911	

Note: ^a^KSA: Kingdom of Saudi Arabia; ^b^HCP: Self-identified health care professional; ^c^Media: Traditional media and journalists (including TV/news websites); ^d^COVID-19: Coronavirus disease 2019; and ^e^Other types of vaccines: Human papillomavirus, influenza, rotavirus, measles, poliovirus, and rabies vaccination.

Using Kendall's Tau bivariate correlations, the average mini-DISCERN scores were significantly and negatively correlated with the weighted Likes (τb correlation coefficient = −0.193, *P* = 0.004), weighted Comments (τb correlation coefficient = −0.152, *P* = 0.025), weighted Saves (τb correlation coefficient = −0.204, *P* = 0.003), and weighted Shares (τb correlation coefficient = −0.289, *P* < 0.001).

### 3.4. Linear regression analysis for the factors correlated with lower mini DISCERN scores

In the linear regression analysis conducted to identify factors associated with lower mini-DISCERN scores (1.00–3.00) as opposed to higher mini-DISCERN scores (3.01–5.00), the model explained 56.2% of the variance (R² = 0.534, Adjusted R² = 0.511). The overall model was statistically significant (F statistic = 23.332, *P* < 0.001). The regression coefficients revealed significant influences from several variables. Notably, the creator category significantly predicted lower mini-DISCERN scores (β = −0.618, t = −8.902, *P* < 0.001), with HCPs having higher scores on average (mean: 4.818 ± 0.726) compared to lay individuals (mean: 2.003 ± 1.641), highlighting the impact of professional background on content quality. Video topic also influenced scores positively (β = 0.179, t = 2.635, *P* = 0.009), with topics like childhood vaccination (mean: 4.510 ± 1.269) associated with higher scores compared to COVID-19 vaccination (mean: 2.542 ± 1.827). Engagement metrics exhibited mixed effects; while weighted Saves showed a positive yet non-significant trend towards higher scores (β = 0.356, t = 1.760, *P* = 0.081), weighted Shares negatively affected the scores (β = −0.206, t = −2.178, *P* = 0.031). However, the weighted Likes and weighted Comments did not significantly impact the mini-DISCERN scores (Table 3).

**Table 3.** Results of linear regression analysis conducted to identify factors associated with lower mini-DISCERN scores.

Model (Dependent Variable: Overall average mini-DISCERN score)	Unstandardized Coefficients		Standardized Coefficients	t	*P-*value
	B	SE^a^	β		
(Constant)	2.132	0.109		19.588	<0.001
Creator category	−0.324	0.036	−0.618	−8.902	<0.001
Video topic	0.074	0.028	0.179	2.635	0.009
Weighted Likes	0.017	0.018	0.131	0.982	0.328
Weighted Comments	−0.303	0.384	−0.165	−0.789	0.432
Weighted Saves	0.284	0.161	0.356	1.760	0.081
Weighted Shares	−0.124	0.057	−0.206	−2.178	0.031

Note: ^a^SE: Standard error.

## 4. Discussion

In the analysis of Arabic TikTok videos on vaccination, we observed critical patterns that reflect both the diversity and specificity of content underlined by the varied sources and topics of these TikTok videos. The presence of possible misleading content in TikTok has been investigated recently in health contexts such as attention-deficit/hyperactivity disorder (ADHD) [76], mental health conditions [77], myopia [72], sinusitis [78], rhinoplasty [79], autism [80], contraception [81], and COVID-19 and its vaccination [33],[41],[82]. While researchers have analyzed TikTok content on vaccination [33],[41],[82], we provide two novel contributions. First, our study is among the first to entail Arabic-language TikTok videos sourced from multiple Arab countries. This effort can help to fill a critical gap in the digital communication literature by addressing the unique cultural and linguistic contexts within Arab-speaking populations. Second, we examined a broad range of vaccination topics, not limiting the scope to specific vaccines or isolated vaccination campaigns.

In our analysis, the prominence of TikTok videos on COVID-19 and childhood vaccination originating from Egypt and KSA, along with a noticeable increase in content volume over time, can be attributed to several factors as follows. First, the high user engagement from Egypt and KSA could be reflective of their large populations and significant digital penetration, which facilitate broader dissemination and consumption of digital content. Second, the focus on COVID-19 and childhood vaccinations in the video content likely reflects persistent global and regional public health priorities. The continuous relevance of COVID-19, fueled by emerging variants and vaccination updates, likely resulted in a high public engagement to this health emergency [83],[84].

Our analysis of TikTok content on vaccination revealed a notable focus on childhood vaccinations. This pattern possibly indicates an attempt by TikTok content creators to resonate with parents, who may be particularly responsive to health-related content concerning their children. The findings from a systematic review by Kubb & Foran, has revealed that parents were avid consumers of online health information yet often struggle with health anxiety and the reliability of the content they encounter [85]. Although we did not assess how parents received this content, we highlight the importance of offering clear and trustworthy health information by social media platforms like TikTok, given the variability in the analyzed content quality. This is needed to help alleviate parental concerns and support more informed health decisions based on previous evidence showing distrust among parents towards TikTok health content which complicates the interaction with essential health information presented on this platform [86]. Finally, the recent increase in video production coincides with TikTok rising popularity and mirrors the global trends [28],[87]. This suggests that TikTok has become an increasingly used tool for disseminating health information as it grows within the digital space. This has been shown in a recent bibliometric analysis by Rejeb *et al*. demonstrating TikTok's ability to deliver accessible and engaging content that is particularly valuable in addressing urgent public health needs [88].

In our analysis of TikTok videos related to vaccination in Arabic, a noteworthy disparity in the number of followers across source categories was observed. Specifically, the “Media” accounts had the highest average follower count, followed by HCPs, and lay individuals. This variation likely reflects broader trends in social media where established media entities naturally amass large number of followers due to their brand recognition and trust built over time. On the other hand, HCPs are expected to have smaller recognition than media organizations, which can invest more resources into maintaining a strong online presence. Lay individuals generally have the smallest reach, reflecting their informal and personal nature of content creation, which may lack the broader appeal of professional content producers. Despite these differences in follower count, our study found no significant variations in the absolute number of engagement metrics across these categories. However, when adjusted for follower count, lay individuals' content was found to have a disproportionately higher engagement. This suggests that while lay individuals have fewer followers, their content may resonate more deeply with their audience. Several hypotheses can be drawn from these observations as follows.

Content from lay individuals often resonates more with audiences on social media due to its perceived relatability, compared to the more refined outputs from media companies and HCPs. This relatability can cause personal stories or anecdotal experiences to feel significantly more impactful than professional advisories or news reports [89]. A study by Sharabati *et al*., suggested that TikTok user satisfaction increases when content encourages self-expression, provides useful information, features a sense of belonging, and aligns with current trends, areas where lay influencers could outshine other content creators such as HCPs [90]. This tendency was supported by a study by Dimitroyannis *et al*., which found that videos on pediatric acute otitis media posted by non-medical influencers on TikTok received more views and shares than those created by HCPs, highlighting the appeal of lay content in engaging viewers [91]. Additionally, in the context of COVID-19 and its vaccination, Baghdadi *et al*. demonstrated that while videos containing significant misinformation were viewed less frequently, they tended to engage viewers more deeply, albeit this was not statistically significant [63]. Moreover, content with lower quality garnered higher engagement metrics normalized by the number of followers in a study by Abdaljaleel *et al*., which highlighted the spread of measles-rubella vaccine misinformation in Jordan [62]. Furthermore, Boatman *et al*. showed that anti-HPV vaccine TikTok videos received more user interactions [92]. However, this trend is not universally observed across all platforms or topics. A contrasting pattern emerged on Instagram, where a study found that posts from nutrition professionals were perceived by young adults as more authentic and trustworthy compared to those from social media influencers [93]. Additionally, Yang *et al*. demonstrated in their analysis of COVID-19 vaccine videos on TikTok that pro-vaccine content was more prevalent than anti-vaccine content without any significant correlation with user engagement metrics [94]. This discrepancy suggests that the platform, context, and subject matter significantly influence how users perceive and engage with social media content.

The higher engagement observed in videos from lay individuals on TikTok in this study can also be partly explained by the platform's algorithms, which tend to favor content that quickly attracts viewer interactions—a common feature of videos by lay individuals who often use emotional or sensational content to gain attention [95]. This phenomenon is supported by psychological insights into social media behavior, where emotionally charged content is more engaging and likely to be interacted with [96].

The phenomenon of higher engagement with lower-quality content can also be attributed to the “echo chamber” effect, where social users are more likely to interact with information that reinforces their pre-existing beliefs [61]. This is particularly evident with content from lay individuals, who often tailor their content to resonate with specific community sentiments or group biases, enhancing engagement through either controversy or validation of shared views [97],[98]. The “echo chamber” effect is a well-documented phenomenon in digital communication creating a feedback loop that limits exposure to diverse perspectives on a specific content [61],[99]. This dynamic is reinforced by social media algorithms, which prioritize engagement by promoting content based on a user's past behavior and interests [100],[101]. As a result, social media users are often exposed to homogenous information that confirms their biases, while opposing viewpoints are ignored creating a vicious circle that is difficult to break [102],[103].

On social media platforms like TikTok, “echo chambers” can contribute to the spread of misinformation [56],[104]. In the context of vaccine hesitancy, “echo chambers” can lead to the persistence of misinformation [105]–[107]. Considering the observation that TikTok algorithms are designed to maximize engagement through emotionally charged content, exacerbation of “echo chambers” can be expected with spread of low-quality, highly engaging content over scientifically accurate content [57],[108]–[110].

In this study, the analyzed TikTok content pertaining to COVID-19 vaccination in Arabic was assessed as being of significantly lower quality compared to videos on childhood vaccination, general vaccination, and other vaccine types. This discrepancy is likely due to the dynamic and frequently updated nature of COVID-19 information, which has become a fertile ground for misinformation given its global impact and the rapid evolution of scientific insights into the virus [111],[112]. This issue is particularly critical in Arab countries of the Middle East, where a notably high level of hesitancy towards COVID-19 vaccines was reported compared to other regions worldwide [113],[114]. In contrast, the analyzed TikTok videos on topics like childhood vaccination, which are based on long-standing public health practices with a broad community consensus on its benefits, received higher mini-DISCERN scores. This variation reflects the nature of vaccine hesitancy as a context- and type-specific phenomenon, highlighting the complex interplay of various factors that would shape community trust and attitude to get vaccinated [115],[116].

In this study, we utilized linear regression analysis to explore the possible factors that could be associated with varying quality of TikTok content as reflected in the mini-DISCERN scores. A key finding was the significant impact of the creator's background on content quality. The videos produced by HCPs consistently achieved higher scores compared to those created by lay individuals. This disparity likely reflects the depth of knowledge and adherence to evidence typical of HCPs. Supporting this, research by Dimitroyannis *et al*. specifically analyzed the quality of pediatric acute otitis media videos on TikTok, demonstrating that physicians typically produced content that was not only more beneficial but also of higher quality compared to that of non-medical influencers [91]. Further supporting the importance of credible sources, another study by the same researchers highlighted that most sinusitis-related videos posted by non-medical influencers were inaccurate, despite being presented as medical advice or educational content [78]. Furthermore, a similar pattern of a higher TikTok content quality by HCPs compared to lay individuals was observed by Abdaljaleel *et al*. in the context of measles-rubella vaccination [62]. Moreover, a study by Al-Rawi & Kiana on how frontline HCPs utilized TikTok during the COVID-19 pandemic relates closely to our findings regarding the dissemination of health information on the platform [117]. Similar to our study, which highlighted the significant role of HCPs in providing high-quality content on TikTok, Al-Rawi & Kiana emphasized the enhanced public presence of HCPs on social media and their key role in educating the public about COVID-19, with the use of TikTok demonstrating capacity to distribute vital health information effectively [117]. These consistent findings across studies suggest the necessity for a comprehensive approach to manage health content on social media platforms involving collaboration among policymakers, HCPs, and the platforms [118]–[120]. There is a need to enhance the presence and engagement of HCPs on social media to ensure the dissemination of accurate and reliable health information in these platforms including TikTok [121]–[123]. This is particularly relevant in critical health areas, including vaccination and cancer prevention [124]–[126].

From a policy perspective, the findings of this study highlighted the importance of strengthening social media platforms' ability to regulate misinformation effectively while balancing the principle of free speech. There is a clear need for policies that ensure accurate information is prioritized, especially in public health contexts. A study by van Kampen *et al*. highlighted the inadequacies in TikTok fact-checking and misinformation policies, revealing that the platform struggles to effectively detect misinformation, with some misleading posts remaining visible for several months [43]. This emphasizes the critical need for TikTok to strengthen its misinformation control policy to ensure the reliability of the information disseminated. Finally, longitudinal research could provide deeper insights into the impact of public health messaging on behaviors over time, especially during health crises like pandemics. This approach should also apply to other public health issues discussed on TikTok, such as vaping, to effectively utilize the platform reach in promoting informed decision-making [127].

Several limitations inherent in our study methodology warrant consideration for accurate interpretation of the findings and future research directions. The reliance on a pre-defined set of keywords and search strategies may have constrained the scope of content retrieved, potentially omitting relevant videos that did not use these specific keywords, which would limit the generalizability of our results across all Arabic-language vaccination content on TikTok. Importantly, we did not investigate the use of modified or alternative keywords that could be used to evade content moderation on TikTok. This evasive strategy has been highlighted by Dobbs *et al*. in the context of promoting e-cigarettes to elude detection by TikTok administrators [128]. Future research should entail incorporating strategies to detect these evasion techniques, which could provide further insights into how misinformation persists on social media platforms despite moderation efforts. Additionally, the cross-sectional design of the study is another caveat since this design enables the study of a momentary snapshot without reflecting changes in TikTok content or user engagement with the videos over time. Moreover, the use of the mini-DISCERN instrument for assessing content quality introduces subjectivity, as different raters might interpret content variably, despite high inter-rater reliability. The modification of the original DISCERN tool and the broad categorization of content creators could also simplify the analysis at the expense of capturing the full complexity of content creators' impact on information dissemination. Researchers could enhance their approach by expanding the keyword list, incorporating longitudinal designs, and employing more diverse and comprehensive assessment tools to gain a deeper understanding of health information dissemination on TikTok and its effects on public health outcomes.

Finally, beyond the aforementioned methodological limitations, caveats pertinent to TikTok-specific features should also be acknowledged. The TikTok algorithms could play a significant role in determining the visibility of content, often promoting sensational or emotionally charged videos over more factual or balanced content. This algorithmic bias was not considered in our study and may have influenced engagement metrics, thereby affecting the visibility of high-quality health information. Additionally, TikTok trends can significantly drive viewers' engagement, irrespective of content quality. We did not consider the role of such trends, which could skew engagement metrics in favor of popular but less reliable content.

## 5. Conclusions

We demonstrated the role of TikTok to spread health information among Arabic-speaking populations, specifically regarding vaccination. We found that HCPs and media outlets produce higher quality content, as evidenced by mini-DISCERN scores, than lay individuals. This highlighted the importance of expert-driven content in ensuring the accuracy and reliability of information shared on social media. However, our findings also revealed a concerning trend with videos from lay individuals suffering from lower quality and received a higher engagement. This suggests that TikTok algorithms may preferentially promote sensational or emotionally charged content, which could lead to the spread of misinformation. Additionally, our results indicated that content on well-established topics, such as childhood vaccination, generally received higher mDISCERN scores compared to newer topics like COVID-19 vaccination, which were more prone to misinformation. The evolving nature of the science around COVID-19 vaccines, coupled with public uncertainty, may have created space for misinformation to circulate more readily. Given these insights, it is clear that strategic interventions are needed to improve the quality of health information on TikTok, particularly for emerging health topics. Enhancing the visibility and reach of content produced by verified sources is a crucial step. Moreover, social media platforms like TikTok should consider adjusting their algorithms to prioritize the reliability of information, especially on critical health topics, to effectively mitigate misinformation and support public health efforts. While TikTok has potential as a public health communication tool, its effectiveness is limited by algorithmic biases that favor engagement over accuracy, which highlights the need for comprehensive strategies to utilize this platform for positive public health outcomes. Our findings suggested that public health policies should focus on enhancing the production of high-quality, expert-driven TikTok content. Additionally, public health policies are recommended to create a regulatory framework that encourages TikTok to play a more active role in restraining misinformation. Public health authorities could develop guidelines for the creation of digital content and work with TikTok to create verification mechanisms that make trustworthy content more prominent.

## Use of AI tools declaration

The authors declare they have not used Artificial Intelligence (AI) tools in the creation of this article.
